# Knowledge, Attitudes, and Practices Concerning COVID-19 in Bangladesh: A Qualitative Study of Patients With Chronic Illnesses

**DOI:** 10.3389/fpubh.2021.628623

**Published:** 2021-12-22

**Authors:** Shaharior Rahman Razu, Nishana Afrin Nishu, Md. Fajlay Rabbi, Ashis Talukder, Paul R. Ward

**Affiliations:** ^1^Sociology Discipline, Khulna University, Khulna, Bangladesh; ^2^Institute of Education and Research, Khulna University, Khulna, Bangladesh; ^3^Statistics Discipline, Khulna University, Khulna, Bangladesh; ^4^College of Medicine & Public Health, Flinders University, Adelaide, SA, Australia

**Keywords:** attitude, Bangladesh, chronic health illnesses, COVID-19, knowledge, practice

## Abstract

The novel coronavirus disease (COVID-19) has posed a serious risk with pre-existing health conditions. This study was conducted to understand the knowledge, attitude, and practices concerning COVID-19 among patients with chronic illnesses in Bangladesh during the pandemic. The study was conducted in Khulna city of Bangladesh following a qualitative research design. We employed telephone interviews to collect data from 40 participants with four common pre-existing chronic illnesses (diabetes, hypertension, respiratory/asthma, and heart disease). Findings show that the majority of the participants had a moderate level of knowledge and an overall positive attitude regarding COVID-19 but appropriate safety practices were often ignored as the pandemic grows older. We also observed that the knowledge, attitude, and practice regarding COVID-19 varied based on age, marital status, education, social class, and rural/urban residence. We concluded that improving medical advice/support, promotion of awareness through mass media, strict monitoring of protective measures and subsidies from the government, and self-consciousness could be effective strategies to mitigate the transmission of the disease and reduce risks for patients with chronic illness in Bangladesh during the COVID-19 pandemic.

## Introduction

The coronavirus disease-2019 (COVID-19) pandemic has disrupted every aspect of human life making people vulnerable to the disease (1). To curb the spread of the coronavirus infection, national and partial closures have already been implemented in most countries around the world. At the same time, countries are following protective safety measures, such as hygiene practices and social distancing, suggested by health experts. Bangladesh, one of the most densely populated countries of the world with a population of 165.2 million, has been highly susceptible to COVID-19 since no proven vaccine or medicine is available for the disease right now (2, 3). There have been 417,475 confirmed COVID-19 cases and 6,036 confirmed deaths owing to the disease in this country so far, and the number is still counting (4). The pandemic can have a serious impact on the country due to its large population size, vulnerable economy, and weak healthcare system. The healthcare system of Bangladesh is still not well-prepared to face this health emergency (5).

Due to the high transmissibility and unavailability of vaccines at this moment, COVID-19 has become a serious concern for people with chronic illness (3, 4). Under the lockdown situation, these people are facing difficulties in taking regular checkups and emergency services making them more vulnerable. Chronic illnesses, such as heart diseases, diabetes, asthma, hypertension, cancer, and HIV, are the major leading causes of death in almost all countries around the world (6). Although these diseases are common worldwide, the burden of such diseases is much higher in developing countries than the developed ones (7).

Knowledge regarding the disease, attitude toward it, and the practices concerning COVID-19 can play a significant role under the circumstance. Knowledge regarding the disease refers to the belief of an individual about the symptoms, treatments, causes, and prevention of the disease (8). While knowledge may control diseases and save lives (9), in many cases, wrong knowledge or misconceptions may endanger the lives of people. Attitudes are also important in handling diseases as positive attitudes make relaxation among people while negative attitudes create anxiety, depression, insomnia, and irritability (10). Practices, on the other hand, are built on knowledge and attitudes ought to be evaluated during pandemics as this will enable policymakers to find out the real scenario during the COVID-19 pandemic (11).

It is evident that the study of knowledge, attitude, and practices concerning the disease among at-risk populations is useful to prevent, control, and mitigate infections during epidemics (5, 12). Most of the existing literature shows the scenario of different communities from the general population, but there is very limited knowledge on the patients with chronic illnesses despite their high vulnerability to COVID-19 (6). Considering this knowledge gap, the main aim of the present study was to identify the knowledge, attitude, and practices concerning COVID-19 among patients with chronic illnesses in Bangladesh through a qualitative study. The findings of this study will help to formulate and revise, and policies concerning interventions aimed at reducing transmission, spread, and contracting COVID-19.

## Analytical Framework

For analyzing the knowledge, attitudes, and practices of patients with chronic illnesses regarding COVID-19 in Bangladesh, we used the knowledge, attitudes, and practices (KAP) model in our study. The theory was first introduced in the 1960's to explain human health change behavior (13). The model has been classified into three consecutive processes, i.e., knowledge, general attitudes, and adoption (practices) of behaviors called KAP theory. According to this theory, there is a progressive relationship among knowledge, attitudes, and behavior as follows: knowledge is the foundation of behavioral change, while belief and attitudes are the driving force of behavioral change. The basic presumption is that one's health promotion and effective illness management are linked with KAP level. On the other hand, poor health and maladaptive disease preventive behavior are associated with KAP deficiency (14). This way we assume that the knowledge, attitudes, and practices of patients with chronic illnesses concerning COVID-19 can effectively increase or decrease their awareness, positive attitudes, and behaviors influencing the disease outcome. We, therefore, took KAP as the analytical model considering its relevance to the assessment of the knowledge, attitudes, and practices of patients with chronic illnesses concerning COVID-19 in Bangladesh.

## Methods and Materials

We conducted a qualitative study focusing on the narrative of patients with pre-existing chronic illnesses to bring out rich information on the topic of our study (15). The research was performed in Khulna city of Bangladesh from May 2020 to September 2020. Given the risks of contacting COVID-19 with face-to-face interviews, we chose telephone interviews to collect data from our participants. Instead of population statistics, our sampling involved the particularly vulnerable group of patients with chronic illnesses during the pandemic as we selected the respondents purposively. The contact details of the participants were collected from the registration records of different clinics and health centers. We called the patients with chronic illnesses who visited medical practitioners in those clinics and health centers previously over the phone and briefed them about our research initially. Those who agreed to participate in our study were contacted further for data collection.

We interviewed a total of 40 patients with four types of common chronic illnesses (diabetes, hypertension, asthma, and heart disease). Ten participants with each of these chronic illnesses were selected for interviews in this study. We used a semi-structured interview guide developed through a rigorous analysis of the previous literature on similar topics. Starting from the age of 18, we included patients from each of the four chronic illness categories where respondents of different ages, sex, religion, residence, marital status, and social classes participated to ensure maximum diversification of information (Table 1). While conducting qualitative research, telephone interviews can often be difficult to bring out narrative data. So we employed effective strategies based on lessons learned from previous studies that followed the same technique (16, 17). We cultivated rapport, maintained regular connection, incorporated concerns, and acknowledged the contribution of our participants to ensure maximum effort and overcome the adversities that might limit the scope of this study during the 4-month of data collection. The duration of each interview varied based on the convenience of the respondents and the data collectors through an average session would last between 30 and 40 min. As it is often difficult to take the interview over the phone for a very long time and to enter into a deep conversation within a very short time, we carefully maneuvered this issue throughout data collection. Potential inquisitive questions and probes were used for further understanding of the reality experienced by the participants. We included a set of questions for our interviews in this study and provided this as Supplementary Material along with this manuscript. Authors, SRR, NAN, and MFR, conducted the interviews and collected data through multiple sessions and with the convenience of the participants.

**Table 1:** Sociodemographic profile of the participants.

**SI**.	Name*	**Age**	**Sex**	**Marital status**	**Education**	**Residence**	**Social class**	**Illness type**
1	Shamim	52	M	Married	Tertiary	Urban	Upper	Diabetic
2	Rina	55	F	Married	Secondary	Urban	Lower	Heart
3	Sumon	32	M	Unmarried	Primary	Rural	Middle	Diabetic
4	Taher	45	M	Married	Secondary	Urban	Lower	Hypertension
5	Asha	29	F	Unmarried	Secondary	Urban	Upper	Asthma
6	Mitu	33	F	Unmarried	Illiterate	Rural	Lower	Hypertension
7	Shirina	45	F	Married	Primary	Rural	Middle	Diabetic
8	Shila	26	F	Unmarried	Tertiary	Urban	Upper	Heart
9	Rayhan	28	M	Unmarried	Secondary	Urban	Middle	Asthma
10	Alam	65	M	Married	Primary	Rural	Lower	Hypertension
11	Sabbir	56	M	Married	Primary	Urban	Middle	Heart
12	Naima	60	F	Married	Primary	Rural	Lower	Heart
13	Shawon	25	F	Unmarried	Secondary	Urban	Upper	Asthma
14	Kawsar	40	M	Married	Secondary	Urban	Upper	Diabetic
15	Titu	70	M	Married	Illiterate	Rural	Middle	Hypertension
16	Mila	56	F	Married	Primary	Rural	Lower	Asthma
17	Ariyan	29	M	Unmarried	Tertiary	Urban	Upper	Hypertension
18	Munni	45	F	Married	Primary	Rural	Middle	Diabetic
19	Kulsum	25	F	Unmarried	Tertiary	Urban	Upper	Heart
20	Shahajul	66	M	Married	Secondary	Urban	Middle	Diabetic
21	Shamim	26	M	Unmarried	Primary	Rural	Middle	Diabetic
22	Jamal	55	M	Married	Secondary	Urban	Middle	Heart
23	Soheli	60	F	Married	Primary	Rural	Lower	Hypertension
24	Sarif	25	M	Unmarried	Secondary	Urban	Middle	Hypertension
25	Jahir	29	F	Unmarried	Tertiary	Urban	Upper	Asthma
26	Jony	31	M	Married	Illiterate	Rural	Lower	Asthma
27	Shirina	45	F	Married	Illiterate	Rural	Middle	Diabetic
28	Safikul	26	M	Unmarried	Secondary	Urban	Middle	Heart
29	Rani	28	F	Unmarried	Secondary	Urban	Upper	Asthma
30	Atahar	65	M	Married	Illiterate	Rural	Lower	Hypertension
31	Shiuli	33	F	Unmarried	Primary	Urban	Middle	Heart
32	Azhar	60	M	Married	Illiterate	Rural	Middle	Heart
33	Firoz	25	M	Unmarried	Secondary	Urban	Upper	Asthma
34	Munni	40	F	Married	Tertiary	Urban	Middle	Diabetic
35	Rafiul	35	M	Unmarried	Primary	Rural	Middle	Hypertension
36	Moni	56	F	Married	Secondary	Rural	Upper	Asthma
37	Jafar	29	M	Unmarried	Secondary	Urban	Upper	Diabetic
38	Munni	45	F	Married	Primary	Rural	Middle	Heart
39	Kulsum	25	F	Unmarried	Tertiary	Urban	Middle	Asthma
40	Selim	66	M	Married	Secondary	Rural	Upper	Hypertension

* *Identities used in this table are pseudonyms*.

### Data Analysis

All the data collected were recorded in information sheets, and audio has taped simultaneously for analyses. The transcribed data were coded and subsequently categorized for thematic analysis. SRR and NAN independently coded the data from the transcript developing a code structure initially, which was later finalized with the consent of the other authors. Level of knowledge, the pattern of attitude, and practices of the participants were measured focusing on accuracy, meaning, phrase, context, clause or concept, and frequency and intensity of comments as we ranked them into high/positive, moderate, and low/negative, categories for reference. Apart from using a qualitative data analysis software QDA Miner, we went through the records line-by-line as well. We selected the most important and repetitive quotes to represent the selected themes as five overarching themes emerged from our analyses—level of knowledge, sources of information, attitude and beliefs, hygiene practices and the use of protective equipment, and social distancing.

### Ethical Considerations

We maintained strict ethical standards for conducting this study. We took informed consent from the participants before data collection as they were briefed about the subject matter of the study. The participants were assured that all the information they have provided will be kept confidential and that their responses will be used only for academic purposes. We used pseudonyms to keep the anonymity of all the participants in our study. We also obtained approval from the Ethical Clearance Committee of Khulna University for conducting this study.

## Results

### Level of Knowledge

We found that the level of knowledge among people about COVID-19 was moderate in our study. However, literate participants had sophisticated knowledge about COVID-19 as they had access to different sources, such as television, radio, and the internet. On the other hand, people who did not have sufficient education or had no access to media developed their knowledge from their surroundings, such as friends, family, or other contacts.

One of the participants with asthma with relatively higher education expressed, “*COVID- 19 is not a serious disease. We may control this virus by following the instructions of the government. Nevertheless, I have known that high temperature can kill the COVID-19 virus though I'm confused about it. There is mixed literature about the link between temperature and the spread. I hope, the outbreak of this virus will decrease after the availability of the Corona vaccine as most of the countries are not going for herd immunity.”*

Such statements imply a sophisticated knowledge of participants with higher education under the context although not all participants had so that much knowledge regarding the disease. We also observed that COVID-19 was regarded as a major threat at the beginning of the pandemic, but as time passed, the fear of COVID-19 decreased significantly. By the end of our data collection period, most of the participants were not worried about the disease anymore and were ready to deal with the implications of the pandemic.

Another male participant with diabetes stated that “*I am not worried at all! Coronavirus is a serious disease. but not everyone dies from it. Since COVID-19 is transmitted through respiratory droplets, maintain social distances and wearing protective equipment can help us prevent the spread of this disease.”*

However, there were also misconceptions about the virus and its remedies. Though participants from an urban background, with a higher level of education and upper social class, had good knowledge about the disease and those who have not adequate education, lived in rural areas and from lower social class had a certain misunderstanding about the disease.

One of the participants with hypertension and a low-level education stated, “*Why should I bother about coronavirus! The medicine (vaccine) is available now. Now we should go back to our normal life.”* Another participant with heart disease expressed, “*I have seen different types of information in the social media. While some say the disease has symptoms, the others say it doesn't have any. Some people are saying high temperature kills the virus, while others nullified it. It's difficult to understand what is right and what is wrong these days.”* These participants highlighted the difficulties of “understanding” the risk factors for COVID-19 and optimal strategies for preventing transmission due to the confusing and often mixed messages they received from different information sources.

### Sources of Information

Under the lockdown situation, most of the participants with a higher level of education and higher social classes informed that they would stay indoors and gathered information regarding COVID-19 from various sources, such as national and international dailies, TV channels, and the internet.

One of our participants shared, “*One day I was listening to the news on television. I suddenly got informed about COVID-19 then I asked my friends about it. Gradually I developed some knowledge about it.”*

However, sources varied for people from different backgrounds. When we asked a female garment worker with a hypertension condition, the 33-year-old replied, “*I do not have time to watch television. I heard of it for the first time when my fellow workers shared this news (COVID-19) sharing the news about COVID-19. I remember our manager ordered us to maintain social distancing and ordered that we should wash our hands frequently and we must have to use a face mask while working in the industry.”*

The source of knowledge for the participants also differed based on geography. Participants who lived in city areas had access to the internet while participants who lived in rural areas did not have that. One of our participants from the city area shared, “*I came to know about COVID-19 from Facebook first. At first, I did not realize that it was that fatal and contagious. But after some time, I came to know about further details that it is so far the most contagious disease where the death rate is not very alarming but the rate of infection is very much shocking.”*

### Attitudes and Beliefs

Although a large number of people in Bangladesh have a low level of education and awareness regarding health matters, to our surprise, we observed that overall, the participants had a positive attitude toward COVID-19 in general. For example, they were supportive to people who contracted the disease or would not generally stigmatize someone for being COVID-19 positive at this stage. This may be due to various factors, such as widespread media circulation and awareness programs from the government during the pandemic.

A female participant with hypertension explained, “*People need to be more supportive during the lockdowns. Mental health is equally important as it affects our immunity system. I am ready to help to maintain social distancing.”* While another male participant with asthma from a rural background opined, “*Although COVID-19 is a contagious disease, a patient infected with it cannot be blamed. We should work together and support him/her during this distress.”*

However, things were not the same in the initial phase of the pandemic, when people were obviously frightened and were unwilling to take the risk of contracting COVID-19. As months passed, they started to accept the risk of contracting COVID-19, partly to enable “normal” functioning in life and partly because of their perceptions of low mortality risks associated with COVID-19. Even there is still some prejudice against diseases, such as HIV, jaundice, cholera, malaria, and typhoid, in Bangladesh as some people consider these diseases as a curse. They often explain the epidemics and infectious diseases from their own supernatural beliefs. Such explanations are more common in rural areas.

There was also a religious element to the perceived risks of contracting COVID-19, which seemed to also be linked to participants with lower education and in lower social classes, “*Real Muslims are not affected by COVID-19. Because they perform ablution during the five daily prayers according to Islamic law. The persons who strictly follow the rules of Islam, can't be affected by this virus.”* Another respondent expressed similarly, “*I think it is a course from God because of our wrong deeds. When the amount of evil deeds increases, such wraths from Allah is inevitable.”* Participants who believed that COVID-19 would not affect “real Muslims”and was an act of God would be less likely to adhere to Government guidelines about social distancing, social isolation, and other risk mitigation strategies.

### Hygiene Practices and the Use of Protective Equipment

Proper hygiene practices are extremely important to control the transmission of COVID-19. However, our participants reported that they followed hygiene practices concerning COVID-19 strictly initially when the pandemic started. As time passed, they started to care less and were less rigid in following practices, such as frequent hand-washing or using sanitizers regularly. This scenario was more common among participants from rural areas. *One* of our respondents from rural background said, “*is extremely difficult to follow each instruction they (government) give. I don't think it's that much necessary to cleanse my hands with sanitizer every single time I touch any object.”*

Aside from following these practices, the use of protective equipment is strongly recommended to keep COVID-19 away. Some participants also reported that though they had the willingness to buy sanitizers, masks, and gloves for protection but they could not pay for them. One of our male participants with diabetes mentioned, “*Sanitizer companies have raised the price of sanitizers and hand-wash products. A one-time mask costs 20 takas (Bangladeshi currency) these days. Is it logical to spare 20 takas for a one-time usable mask? I am not going to do it.”*

Many participants expressed that they were unwilling to use protective equipment due to lack of willingness and discomfort. One of our participants with asthma condition narrated, “*I think it's impossible to wear a mask all the time. Many of these are cheap in quality and the premium ones block so much air that it makes it difficult for me to breathe properly. That's why I have decided to wear a mask only when it's an emergency.”*

A few participants, however, expressed that they are cautious about hygiene maintenance and wearing protective equipment due to their previous experiences. “*I became seriously ill when I had this coronavirus a few weeks back. Since then, I use mask whenever I go outside and avoid touching my eyes, nose, and mouth. I try to cover my nose and mouth with a tissue whenever coughing or sneezing and throw the tissue in the trash after using it. At the same time, frequently I wash my hands with hand sanitizer and soaps. I am trying to pay more attention to my hygiene than usual as I know I am weak (immunocompromised).”*

Most of our participants shared that they have become less interested in taking nutritious foods or vitamin supplements to keep themselves healthy as days passed during the pandemic. They would not exercise or monitor their health regularly although the scenario was quite different at the beginning.

### Social Distancing

The WHO recommends the social distancing of 1 m from one person to another to prevent transmission of the COVID-19 (18). This is why countries have imposed nationwide lockdowns and closed many institutions to avoid public gatherings. From our study, it was revealed that almost all the participants had some knowledge about social distancing, but none would follow the instructions. The reason they mentioned was either obligation or unwillingness as one of our young male participants with hypertension condition shared, “*I have to go to my factory every day. Otherwise, I will lose my job. I know that everyone should keep some distance from one another, but it is quite impossible for me because I have to use public transport each day. No one is following social distancing in this country. Why should and how can I do that alone!”*

Most of the participants agreed to this statement that maintaining social distances was almost impossible for them when a majority in the society is careless about it as narrated by one of our respondents with heart disease, “*I don't think it is possible to maintain social distancing in the country. Look at this huge population! Most of them are unwilling to stay home for a long time. It is impossible to stay home for a long time.”*

Some also pointed the inability of the government to maintain social distancing, as a participant opined, “*The government failed to implement social distancing. You cannot make things work this way. Look at these poor people. They have many dependents in their family and our government simply cannot provide them with daily necessities for even 1 month.”* Another female participant with diabetes expressed her concern saying, “*I have been living in fear of contracting COVID-19 these days. Though I am staying home, the other members of my family are frequently visiting outdoors. People do not even care about social distancing these days. Who knows what they (family members) are bringing home!”*

## Discussion

The objective of this study was to investigate the knowledge, attitude, and practice concerning COVID-19 among the patients with chronic illnesses in Bangladesh. To the best of our knowledge, this is the very first qualitative study using KAP theory in the country, and one of the very few over the globe. While conducting this study, we tried to get the most detailed information from patients with chronic illnesses regarding their experiences, beliefs, and concerns during the pandemic. Corresponding to some of the existing literature, we observed that most of the participants had a moderate level of knowledge about the transmission, symptoms, and prevention of COVID-19 (19–22). Even though the participants had decent knowledge about COVID-19, they practiced less in accordance with their knowledge level. Besides, although the attitude toward COVID-19 was positive in general, misconceptions regarding the disease were also reported by some participants. However, contrary to our findings, some other studies have reported positive attitudes and good practices concerning COVID-19 (18–22).

We found that patients with chronic asthma and heart disease were generally more concerned about COVID-19 compared to the other risk groups in our study. This might be due to the fear of the increased risk of severity for heart and lung conditions (23). Besides, almost all the participants in our study faced treatment difficulties during the COVID-19 period as many doctors would not visit patients during the pandemic. While epidemic outbreaks can cause psychological trauma and negative emotions, such as fear, anxiety, and helplessness, the unavailability of doctors during the COVID-19 pandemic affected the mental state of patients with chronic illnesses (5, 24). We noted that all the participants in our study were more careful about their health during the initial COVID period than pre-COVID time. As time passed, the majority of the respondents started practicing safety behaviors less and would rarely wash their hands with soap or sanitizer. After a few months of the inception of the pandemic, touching face with unwashed hands, shaking, moving outside became very common (6). To maintain risk mitigation behaviors, more regular Government mass-media messaging may be required.

Previous studies also mentioned that to control infectious diseases such as the H1N1 flu outbreak, the assessment of the knowledge, attitude, and practice of people has played a significant role (8, 25–27). Knowledge, attitudes, and practices regarding COVID-19 among the patients with chronic illnesses might play an important role to combat the transmission, especially in countries, such as Bangladesh, where health facilities are poor (1, 28).

The KAP theory suggests that knowledge of people is often derived from their education. Corresponding to this theory, we also observed that the education and knowledge of our participants are interrelated (22). We found that participants with higher social status and living in urban areas had higher knowledge levels and followed the safety instructions recommended by the Ministry of Health and Family Welfare of Bangladesh and WHO more accurately than the others. Around two-thirds of the total population in Bangladesh live in rural areas while most of them are poor (2, 24). While these people had some basic knowledge, they were often unwilling to practice the health instructions properly due to work-related and financial reasons (6). Besides financial issues, we also recorded that religion was linked with knowledge, attitude, and practices concerning COVID-19. We observed that many orthodox followers of Islam were unwilling to practice social distancing and believed that the COVID-19 pandemic is a curse from God and will not affect the believers (15). Research is required to understand if this is similar in other predominantly Muslim countries, and indeed, in other orthodox followers of other religions around the world. Targeted public health information is required to work with religious communities to reduce the risks of transmission of COVID-19.

Knowledge, attitude, and practices of people concerning any health behavior are largely dependent on their sources of information. In line with some existing literature, our study showed that the majority of the participants with a higher level of educated obtained information regarding COVID-19 from mass media such as television, radio, and the internet (6, 15, 19, 22, 29). It is interesting that, more than surrounding people like friends and family or healthcare professionals, mass media played a more important role in spreading awareness on COVID-19 and helped to curb the transmission of the disease (30, 31). In contrast, the participants with low education have reported learning about the virus mostly from people around them (29, 31). We found a majority of the participants to hold an optimistic attitude toward COVID-19 patients and expressed positive ideas to overcome the pandemic in line with some of the previous research (20, 22). We also observed that safe practices were significantly higher among the women, married, educated, and those who live in urban areas (6, 19) while safety practices and use of protective equipment were less common among the unmarried, illiterate, and rural men (19, 22).

## Strengths and Limitations

We acknowledge that our study has certain limitations as we had to conduct the research during the pandemic situation. It was not possible for us to collect data through face-to-face interviews during the series of nationwide lockdowns. We also admit that it is difficult to conduct research using this technique. However, we employed effective interviewing strategies, such as maintenance of regular contacts and recording concerns of the interviewees, over a significant time to overcome the odds. Besides, there may be questions regarding the simplification of results with a few numbers of participants, but we strengthened the rigor of information by the quality of the data in our study. The main strength of this study, however, was its novelty that we employed KAP theory from the qualitative perspective in Bangladesh for the first time in this study. We believe that the findings of this study can provide important insights for policymakers to formulate and improvise awareness programs and strategies to manage the pandemic or similar events in the future.

## Conclusion

Patients with chronic illnesses in Bangladesh have become vulnerable during the COVID-19 pandemic. The findings of this study revealed that the patients with chronic illnesses had moderate knowledge and a positive attitude toward COVID-19, but their safety practices were weak. Although the government has already taken several steps to mitigate the spread of the diseases, it has been difficult to ensure proper safety practices due to different socioeconomic issues. Applying the KAP theory, we tried to how the participants' knowledge, attitude, and practices depend on their level of awareness. Owing to these facts, health education programs and policy interventions are necessary to ensure the health and well-being of patients with chronic illnesses right now. Under this circumstance, the above findings have some important policy implications. First, awareness programs on mass media should be emphasized more under the lockdown situation. Second, specialized and emergency medical services should be ensured for the patients with chronic illnesses. Third, the government needs to impose safety practices more strictly. Special subsidies on hygiene products and personal protective equipment can be provided to make it more available for people. Finally, we suggest that patients with chronic illnesses be prioritized when the vaccines are available. Preferably, it should be provided free of charge to the vulnerable groups who are at high risk at present. While implications of this study will help policymakers and researchers, we recommend further research on this topic to get a comprehensive idea of the knowledge, attitude, and practices concerning COVID-19 in Bangladesh.

## Data Availability Statement

The original contributions presented in the study are included in the article/Supplementary Material, further inquiries can be directed to the corresponding author.

## Ethics Statement

The studies involving human participants were reviewed and approved by Ethical Clearance Committee, Khulna University. Written informed consent for participation was not required for this study in accordance with the national legislation and the institutional requirements.

## Author Contributions

All authors listed have made a substantial, direct, and intellectual contribution to the work and approved it for publication.

## Conflict of Interest

The authors declare that the research was conducted in the absence of any commercial or financial relationships that could be construed as a potential conflict of interest.

## Publisher's Note

All claims expressed in this article are solely those of the authors and do not necessarily represent those of their affiliated organizations, or those of the publisher, the editors and the reviewers. Any product that may be evaluated in this article, or claim that may be made by its manufacturer, is not guaranteed or endorsed by the publisher.
