# Supplementary material for: Knowledge, Attitudes, and Practices Concerning COVID-19 in Bangladesh: A Qualitative Study of Patients With Chronic Illnesses
Source: Front Public Health. 2021 Dec 22;9:628623. doi: 10.3389/fpubh.2021.628623 (PMC8727516; doi:10.3389/fpubh.2021.628623)
Supplement: Supplementary file 1 [file Table_1.DOCX]

**Supplement Table**

| Questions | | Response |
| --- | --- | --- |
| **Knowledge** | | |
| K1 | Have you ever heard of COVID-19? |  |
| K2 | Where did you learn about COVID-19? |  |
| K3 | How does COVID-19 spread? |  |
| K4 | What are the symptoms of COVID-19? |  |
| K5 | Why is COVID-19 considered to be a serious disease? |  |
| K6 | Which people are more vulnerable to COVID-19? |  |
| K7 | How to test if someone is COVID-19 positive? |  |
| K8 | How can we reduce the risk of contracting COVID-19 through using protective equipment, maintaining personal hygiene and social distancing? |  |
| K9 | What should a personal do if tested COVID-19 positive? |  |
| **Attitude** | | |
| A1 | Where do you believe COVID-19 come from? |  |
| A2 | What are your religion say about infectious diseases such as COVID-19? |  |
| A3 | Do you think people acquire COVID-19 due to their deeds? |  |
| A4 | Would you allow a COVID-19 patient to stay in your house if needed? |  |
| A5 | Should a person with COVID-19 be allowed to roam freely? |  |
| A6 | Are you willing to support a person financially with COVID-19 if required? |  |
| A7 | Are you willing to help a person with COVID-19 in emergency (calling for medical helps, admitting to hospital etc.) if needed? |  |
| A8 | What are your perceptions on the government tackling COVID-19 Bangladesh? |  |
| A9 | What are your thoughts a vaccine for COVID-19? |  |
| **Practice** | |  |
| P1 | Do you wash hands frequently and keep your personal hygiene a priority during the COVID-19 pandemic? |  |
| P2 | When and how frequently do you use protective equipment such as masks and gloves when visiting outdoors? |  |
| P3 | How do you maintain social distancing to avoid contracting and spreading COVID-19? |  |
| P4 | Do you always cover your **mouth and nose** with a tissue when you cough or sneeze? |  |
| P5 | Do you exercise regularly and/or take vitamin supplements/other nutritious foods to boost up your immunity? |  |
| P6 | Do you monitor your health regularly during COVID-19? |  |
